# Time course of the response to ACTH in pig: biological and transcriptomic study

**DOI:** 10.1186/s12864-015-2118-8

**Published:** 2015-11-17

**Authors:** Valérie Sautron, Elena Terenina, Laure Gress, Yannick Lippi, Yvon Billon, Catherine Larzul, Laurence Liaubet, Nathalie Villa-Vialaneix, Pierre Mormède

**Affiliations:** INRA, UMR 1388 Génétique, Physiologie et Systèmes d’Elevage, Castanet-Tolosan, F-31326 France; Université de Toulouse INPT ENSAT, UMR 1388 Génétique, Physiologie et Systèmes d’Elevage, Castanet-Tolosan, F-31326 France; Université de Toulouse INPT ENVT, UMR 1388 Génétique, Physiologie et Systèmes d’Elevage, Toulouse, F-31076 France; INRA, UMR 1331 ToxAlim, Toulouse, F-31027 France; INRA, UE 1372 GenESI, Surgeres, F-17700 France; INRA, UR 0875 MIAT Mathématiques et Informatique Appliquées de Toulouse, Castanet-Tolosan, F-31326 France

**Keywords:** Stress, Hypothalamic-pituitary-adrenal (HPA) axis, Cortisol, Time-course, Systems biology, Microarray, Pig

## Abstract

**Background:**

HPA axis plays a major role in physiological homeostasis. It is also involved in stress and adaptive response to the environment. In farm animals in general and specifically in pigs, breeding strategies have highly favored production traits such as lean growth rate, feed efficiency and prolificacy at the cost of robustness. On the hypothesis that the HPA axis could contribute to the trade-off between robustness and production traits, we have designed this experiment to explore individual variation in the biological response to the main stress hormone, cortisol, in pigs. We used ACTH injections to trigger production of cortisol in 120 juvenile Large White (LW) pigs from 28 litters and the kinetics of the response was measured with biological variables and whole blood gene expression at 4 time points. A multilevel statistical analysis was used to take into account the longitudinal aspect of the data.

**Results:**

Cortisol level reached its peak 1 h after ACTH injection. White blood cell composition was modified with a decrease of lymphocytes and monocytes and an increase of granulocytes (*FDR*<0.05). Basal level of cortisol was correlated with birth and weaning weights. Microarray analysis identified 65 unique genes of which expression responded to the injection of ACTH (adjusted *P*<0.05). These genes were classified into 4 clusters with distinctive kinetics in response to ACTH injection. The first cluster identified genes strongly correlated to cortisol and previously reported as being regulated by glucocorticoids. In particular, *DDIT4*, *DUSP1*, *FKBP5*, *IL7R*, *NFKBIA*, *PER1*, *RGS2* and *RHOB* were shown to be connected to each other by the glucocorticoid receptor NR3C1. Most of the differentially expressed genes that encode transcription factors have not been described yet as being important in transcription networks involved in stress response. Their co-expression may mean co-regulation and they could thus provide new patterns of biomarkers of the individual sensitivity to cortisol.

**Conclusions:**

We identified 65 genes as biological markers of HPA axis activation at the gene expression level. These genes might be candidates for a better understanding of the molecular mechanisms of the stress response.

**Electronic supplementary material:**

The online version of this article (doi:10.1186/s12864-015-2118-8) contains supplementary material, which is available to authorized users.

## Background

Farm animals have been highly selected for favorable production traits such as lean growth rate, feed efficiency, and prolificacy in pigs. In parallel their robustness has tended to decrease, as shown by the sensitivity to diseases, locomotor weakness or behavioral problems [1]. The hypothalamic-pituitary-adrenocortical (HPA) axis plays a major role in physiological homeostasis including metabolism, brain function and behavior, the immune system and inflammatory processes. Together with the autonomic nervous system, it is also involved in stress and adaptive responses to environmental challenges. On the basis of available literature, we have hypothesized that the HPA axis could contribute to the trade-off between production and robustness traits, and that genetic variation in HPA axis activity could be used to select more robust animals [2, 3]. HPA axis activity shows a large variation among individuals and genetic influences are well documented [4]. For example, in pigs, the sensitivity of the adrenal cortex to adrenocorticotropic hormone (ACTH) and the production of corticosteroid binding globulin (CBG), the carrier of cortisol in blood are the two main mechanisms responsible for genetic differences in circulating cortisol levels [5, 6]. In a previous paper, Hazard et al. [7] have studied at the gene expression level the molecular mechanisms of genetic differences in the adrenal gland response to ACTH. However little is known about the individual variation in the biological activity of cortisol, the main glucocorticoid hormone, and the genetic mechanisms involved.

Corticosteroid hormones exert their biological actions via two intracellular receptors (gluco- and mineralo-receptor) that, upon activation by their ligand, influence the expression of a large number (several hundreds) of genes in a wide range of cell types [8]. In pigs, glucocorticoid receptor polymorphisms have been described with their functional consequences [9–13]. The present experiment was designed to explore in pigs individual variation in the biological response to cortisol in order to identify possible biomarkers of this sensitivity. To that end, juvenile pigs were submitted to an ACTH challenge to release cortisol and the kinetics of the response was measured with biological variables and with gene expression analysis in blood cells. Taken together with biological information, this approach will serve as an important step to understand HPA axis regulation and will identify key genes involved in signaling pathways relevant to stress responses. The final goal of this work is to develop a strategy for further genetic studies in order to overcome the unfavourable consequences of stress in farm animals.

## Animals and methods

### Animals, treatment and blood sampling

All animal use was performed under European Union and French legislation (directive 201063UE, décret 2013-118). The protocol and procedures were approved by the local (Poitou-Charentes) ethics committee (decision CE2013-1, 21012013). Experimental animals were 120 piglets (63 females and 57 males) randomly selected from 28 litters (4–5 animals per litter) of purebred Large White pigs and produced in 3 successive batches raised 3 weeks apart. They were weaned at 4 weeks and animals from 2–3 litters were mixed at weaning in each post-weaning pen. Experimental animals were not isolated from their littermates. At 6 weeks, each animal was injected in the neck muscles with 222 *μ*g of synthetic 1–24 ACTH (Pepscan Presto BV, Lelystad, The Netherlands) diluted in 1 mL of 0.9 % saline. Injections occurred from 10:00–11:00 AM to avoid nycthemeral variations. Blood samples were collected before the injection (*t*=0) and 1 (*t*=+1), 4 (*t*=+4) and 24 (*t*=+24) hours later. At each time, animals were slightly restrained on their back in such a way that the effect on their stress level can be regarded as insignificant. Two blood samples were then taken by puncture of a jugular vein in Vacutainer ^*Ⓡ*^ tubes with 20 G needles. The whole handling procedure lasted less than 30 sec. One 10 mL tube with lithium heparin was used for chemical biology. After centrifugation (2355 g, 10 min), plasma aliquots were frozen at –80 °C until analysis. One 5 mL tube with EDTA (di-potassium salt) was used for blood cell count and an aliquot (400 *μ*L) was mixed with the same volume of DL buffer (Macherey-Nagel), frozen at –20 °C for 4 h and then at –80 °C until analysis for gene expression.

### Biological analyses

Cortisol was measured by direct automated immunoassay (AIA-1800, Tosoh Bioscience, San Francisco, CA). Glucose and free fatty acid (FFA), were measured by colorimetry with an ABX Pentra 400 clinical chemistry analyzer from Horiba Medical (Grabels, FR). Blood cell counts were measured with a MS-9-5 hematology analyzer from Melet Schloesing Laboratories (Osny, FR), calibrated for pig blood by the manufacturer. Blood cell count variables included: white cells count, proportion of lymphocytes, monocytes and granulocytes, red cells count, percentage of hematocrit, concentration of hemoglobin, red cells width and volume, concentration of platelets and platelets width and volume. The biological variables contained thus 15 variables measured on the 120 pigs. In addition, birth and weaning weights were also measured for each pig. Outlying observations were visually identified and treated as missing data. Missing data were imputed using a *k*-nearest neighbour (*k*=5) imputation (R package **DMwR** [14]). To ensure normality, cortisol, platelets and white cells count were log10 transformed and FFA was transformed using the square-root. Batch effects were removed by aligning the within-batch medians for all measurements.

### RNA extraction and whole blood gene expression analysis

A total RNA isolation and purification was done according to the manufacturer’s instructions using the Nucleospin RNA Blood kit (Macherey-Nagel, France) followed by DNase treatment. The quality of each RNA sample was checked through the Bioanalyser Agilent 2100 (Agilent Technologies, Massy, France) and low-quality RNA preparations were discarded (RIN <8).

#### Microarray description

A porcine microarray GPL16524 (Agilent, 8×60 K) was used to hybridize the RNA samples as previously described [15]. This microarray contained 61,625 spots. Among them, 308 were negative controls and 49 were used for aligning. One probe was duplicated twice on each array. Thus the microarray contained 60,305 unique porcine probes. After quality control, quantile normalization and filtering, 35,429 transcripts were found to be expressed in blood in our experimental conditions.

#### Hybridization protocol

Blood samples from 30 female pigs from only 2 batches were used. Each of the 15 arrays used contained 8 microarrays which corresponded to the 4 observations of two individuals, each from one batch. This design secured the kinetics of the response for each individual and prevented confounding effects between batch and array. After quality control and filtering, 35,419 probes were kept and log2 transformed. Technical biases were handled by aligning the within-array medians for all genes and by a quantile normalization within animal (function **normalize.quantiles** in the R package **preprocessCore** [16]). No missing data were reported. Normalized data were submitted to GEO/NCBI with the GSE71207 accession number.

### Statistical analyses

All analyses were performed with the R software, version 3.1.0 [17].

#### Multilevel approach

A multilevel approach was used to investigate the relationships between the repeated measurements while taking advantage of multivariate approaches. The multilevel approach, as described by Liquet et al. [18], uses a split-up variation coming from the mixed-model framework. Let $X=(x_{\textit {it}}^{k})_{i=1,\ldots,n,\,t\in \{0,+1,+4,+24\},\,k=1,\ldots,p}$ be the (*N*×*p*) observation matrix (biological variables or gene expressions) on *n* animals with 4 times of measurements (*N*=*n*×4). *X* can be split up as:

(1) $$ {}X = \underbrace{X_{..}}_{\textrm{offset term}} + \underbrace{X_{b}}_{\textrm{between-animal deviation}} + \underbrace{X_{w}}_{\textrm{within-animal deviation}}  $$

The matrix *X*_.._ represents the offset term defined as $1_{N}\,x_{..}^{T}$ where 1_*N*_ is a vector of length *N* containing only ones and $x_{..}^{T}= (x_{..}^{1}, \ldots, x_{..}^{p})$ (with $x_{..}^{k} = \frac {1}{N}\sum \limits _{t\in \{0,+1,+4,+24\}}\sum \limits _{i=1}^{n}x_{\textit {it}} ^{k}$). *X*_*b*_ is the between-animal matrix of size (*N*×*p*) defined by concatenating $1_{4}\, x_{\textit {bi}}^{T}$ for each animal into *X*_*b*_ with $x_{\textit {bi}}^{T} = \left (x_{i.}^{1} - x_{..}^{1}, \ldots,x_{i.}^{p} - x_{..}^{p}\right)\left (x_{i.}^{k} = \frac {1}{4}\sum \limits _{t\in \{0,+1,+4,+24\}}x_{\textit {it}}^{k}\right)$. *X*_*w*_=*X*−*X*_*i*._ is the within-animal deviation matrix of size (*N*×*p*) with *X*_*i*._ the matrix defined by concatenating the matrices $1_{4}\, x_{i.}^{T}$ for every animal into *X*_*i*._, with $x_{i.}^{T}=\left (x_{i.}^{1}, \ldots, x_{i.}^{p}\right)$.

By splitting the different parts of the variation in the data while taking into account the repeated measurements on the subjects, the multilevel step allows us to study the effect of different conditions within a subject separately from the variation between subjects. This method is especially relevant when a high between-subject variability is observed in repeated data: multivariate approaches (such as principal components analysis, PCA [19] and partial least square regressions, PLS [20]) can then be performed on *X*_*w*_ to highlight the most relevant correlations between variables in the dataset, independently from individual variations.

#### Statistical analysis of plasma metabolites and hormone

First, all variables were subjected to a one-way ANOVA with repeated measures. *P*-values were adjusted using a Benjamini-Hochberg (BH) approach in order to control the false discovery rate (*FDR*) [21]. Variables with an adjusted *P*-value (*FDR* <0.05) were then subjected to 3 paired *t*-tests to assess the difference between *t*=0 and *t*=+1, between *t*=0 and *t*=+4 and between *t*=0 and *t*=+24. The full list of *P*-values was adjusted using a BH approach.

In addition, the influence of sex on the biological variables was tested using a two-way ANOVA with repeated measures including sex as a variable. *P*-values were adjusted using a BH approach.

Then, variability between individuals before the ACTH injection was studied by performing a PCA on the observations at *t*=0 for variables responding to ACTH and birth and weaning weights. The overall effect of ACTH over time was investigated with a multilevel PCA as previously described.

Cortisol levels at *t*=+1 is the most relevant measure to assess the sensitivity of the adrenals to ACTH. Hence, correlations between biological variables at *t*∈{0,+1,+4,+24} and the level of cortisol at *t*=+1 were investigated using paired *t*-tests. *P*-values were adjusted using a BH approach.

#### Statistical analysis of the transcriptome

All transcripts were subjected to 3 paired *t*-tests to assess the difference in expression between *t*=0 and *t*=+1, between *t*=0 and *t*=+4 and between *t*=0 and *t*=+24. The full list of *P*-values was adjusted using a Bonferroni approach. As the Bonferroni approach exerts a more stringent control than the BH approach, it was used to obtain a narrowed list of the most significantly differentially expressed (DE) transcripts. Transcripts with at least one adjusted *P*-value <0.05 among the three tests were kept. Correlations between the expression levels of different transcripts of the same gene were investigated to highlight genes for which at least 3/4 of the duplicates were significantly DE and had a correlation of at least 0.65 between them. The most significant transcripts per annotated gene were kept and the multilevel approach was used to extract the within-subject deviation matrix for further analysis.

A hierarchical ascending classification (HAC) was performed using the Ward method with an Euclidean distance matrix based on the correlations between genes. This allowed for the characterization of clusters of genes having the same evolution over time. Significance of the difference between time measurements was assessed using 3 paired *t*-tests at the average gene level between *t*=0 and *t*=+1, between *t*=0 and *t*=+4 and between *t*=0 and *t*=+24. *P*-values were adjusted within the clusters using a BH approach.

#### Integration

Relations between the main stress variable, the cortisol, the biological variables and the gene expressions were studied using different approaches.

As for biological data, correlations between gene expression at *t*∈{0,+1,+4,+24} and the level of cortisol at *t*=+1 were investigated. More precisely, the within-subject components of the transcriptomic data and of the cortisol level was extracted using the method described in [18] and Section “Multilevel approach”. Then, a paired *t*-test was performed between the within-subject expression at *t*∈{0,+1,+4,+24} and the within-subject level of cortisol at *t*=+1. The full list of *P*-values was globally adjusted using a BH approach. In order to assess the significativity of change in expression after removing the contribution of cell population changes, a linear mixed model was fitted for every DEG

$$ x_{it} = \beta_{0} + \beta_{1,t} + \beta_{2} (\text{L/G})_{it} + U_{i} + \epsilon_{it} $$

where *x*_*it*_ is the expression of the DEG for the animal number *i* (*i*=1,…,120) and the time step *t* (*t*∈{0,+1,+4,+24}), (L/G)_*it*_ is the lymphocytes/granulocytes ratio for the same experiment and *U*_*i*_ is the individual random effect. Both time step (as a factor) and (L/G)_*it*_ were supposed to have fixed effects on gene expression. Significance of the time effect in this model was checked by testing *β*_1,*t*_=*β*_1,0_ for *t*∈{+1,+4,+24} and correcting *p*-values by time point for multiple tests with a BH approach (*FDR*<0.05). The effect of changes in white cell populations was also assessed by testing *β*_2_=0 and a correction for multiple tests was applied using a BH approach (*FDR*<0.05).

A multilevel PLS, *i.e.*, a PLS performed on the within-subject components of the biological and transcriptomic data, as described in [18] and Section “Multilevel approach”, was used to investigate the overall relationships between biological and transcriptomic data. A sparse version of the PLS (*L*^1^ penalty) as described in [20] was used to select a small subset of variables to explain every axis.

### Sequence annotation

Each cDNA sequence was compared to Refseq_rna mammalian database using the NCBI blastn program (http://blast.ncbi.nlm.nih.gov/Blast.cgi). Resulting hits were sorted out according to their closeness to the pig genome, their coverage and sequence identity. The selected cDNA sequences were submitted to the HUGO (Human Genome Organization) gene nomenclature committee, using their RefSeq IDs (http://genenames.org). Then, HUGO gene symbols or official gene symbol were used as gene names.

### Functional enrichment and pathway analysis

Functional enrichment was performed on each list of clustered genes identified by HAC and on the list of genes of which expression is significatively explained by cell population changes in blood (according to the mixed model described in Section “Integration”). Functional annotation of genes was provided by the BioMart database [22]. To set the statistical enrichment of a particular biological function, a Fisher’s exact test was performed, using the list of genes expressed on the microarray as the reference list of genes. Resulting *P*-values were adjusted for multiple tests using a BH approach. A minimum of 3 genes per gene ontology and a *FDR*<0.05 were necessary to consider a biological function to be enriched.

A pathway is an interconnected arrangement of processes, representing the functional roles of genes in the genome. Functional integration of gene expression, *i.e.*, identification of gene networks, was performed using the ‘Gene Ontology’ database AmiGO (http://amigo.geneontology.org). The significantly up- or down-regulated genes could be assembled into networks using ‘Ingenuity Pathway Analysis’ (http://www.ingenuity.com) under licence. This application provides computational algorithms to identify the enriched biological pathways, functions and biological mechanisms of selected genes and proposes also enriched regulators as transcription factors.

A regulatory network could be constructed with the information provided by the option ‘Upstream Regulator’. This option proposes a list of regulators known to have a significant effect on some of the targeted genes in the input list. Ingenuity also provides computational algorithms to identify and to dynamically generate significant biological networks. Networks are ranked by a score that takes into account the number of focus genes and the size of the networks. This score (− log10(*P*-value)) indicates the probability for genes to be associated in the same network by chance. A higher score means a smaller probability for genes to be observed in the same network by chance. We chose networks displaying direct relationships between genes. Path Designer (an Ingenuity tool) was used to improve the readability of the networks. Nodes added by Ingenuity were discarded when they were not necessary to connect our genes of interest and the resulting network was merged with the regulatory network.

## Results and discussion

### Plasma cortisol, metabolites and hematology

**Fig. 1:**
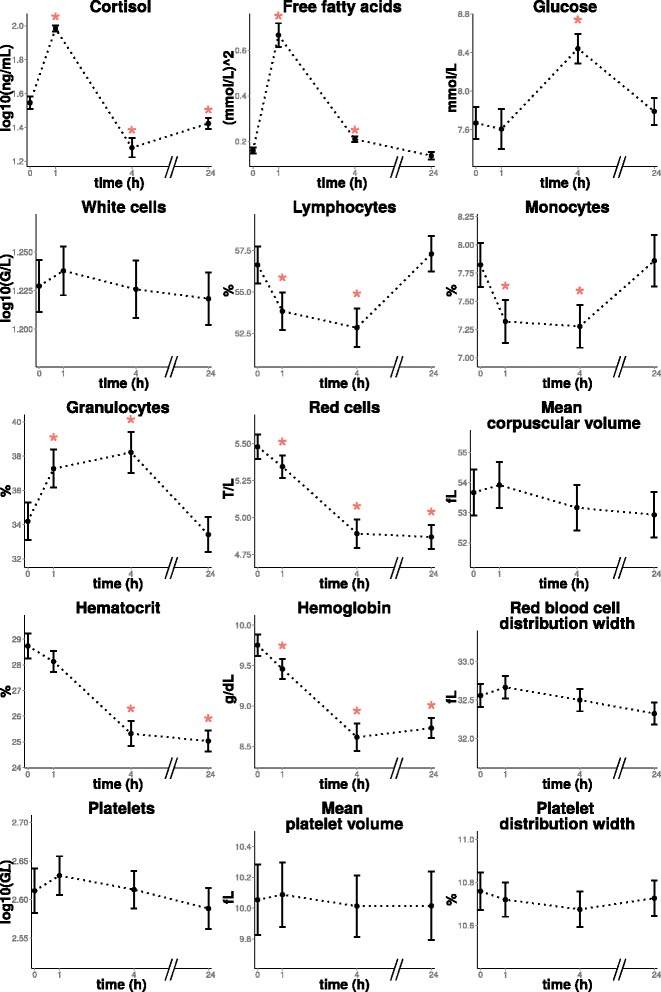
Mean evolution of the biological variables overtime. (*): time measurement at which the expression of the variable is significantly different (*F* *D* *R*<0.05) from the expression at *t*=0. Vertical bars correspond to + and – SEM at each time point

Table 1 shows baseline values of the biological variables, birth weight and weaning weight. Mean evolution over time of the biological variables is shown in Fig. 1. A global effect of time was observed for 14 out of the 15 biological variables (*FDR*<0.05). However, when considered at each time point individually, only 9 variables presented a level significantly different from their basal level for at least one time point (*t*=+1,+4 or +24).

**Table 1:** Reference values (at *t*=0) for the biological variables, birth weight and weaning weight (*n*=120)

	Units	Min	Max	Mean	Sem	*F* value	FDR
Cortisol	log10(ng/mL)	1.04	1.94	1.55	0.04	445.27	<0.01
Free fatty acids	(mmol/L)^2^	0.03	0.39	0.16	0.01	327.69	<0.01
Glucose	mmol/L	5.62	9.84	7.67	0.17	24.36	<0.01
White cells	log10(G/L)	0.96	1.50	1.23	0.02	2.90	0.04
Lymphocytes	%	38.95	69.53	56.63	1.12	50.59	<0.01
Monocytes	%	5.70	11.15	7.82	0.19	15.44	<0.01
Granulocytes	%	18.40	52.70	34.20	1.10	51.96	<0.01
Red cells	T/L	3.88	6.60	5.48	0.08	139.26	<0.01
Mean corpuscular volume	fL	43.10	65.22	53.67	0.77	29.66	<0.01
Hematocrit	%	20.12	36.96	28.74	0.48	165.87	<0.01
Hemoglobin	g/dL	7.70	12.10	9.75	0.14	155.15	<0.01
Red blood cell distribution width	fL	30.25	35.05	32.56	0.15	17.89	<0.01
Platelets	log10(G/L)	1.85	3.13	2.61	0.03	14.07	<0.01
Mean platelet volume	fL	8.00	14.20	10.05	0.23	0.68	0.57
Platelet distribution width	%	9.90	11.90	10.76	0.09	4.08	<0.01
Birth weight	Kg	0.40	2.68	1.50	0.07	NR	NR
Weaning weight	Kg	5.46	16.56	9.4	0.35	NR	NR

*F* value and FDR are for the test of the global time effect on each variable. *NR* non relevant since the measure is the same at all time steps

As expected [23], ACTH induced a strong cortisol response peaking 1 h after injection (*FDR*=1.39*e*^−10^). Plasma cortisol levels at *t*=+1 were 2.7-fold higher than and highly correlated with basal levels (*r*^2^=0.63,*FDR*=1.95*e*^−14^). They were significantly lower than baseline values at *t*=+4 (*FDR*<2.2*e*^−16^) and at *t*=+24 (*FDR*=1.39*e*^−10^), as can be expected from the feedback of cortisol on its own secretion. Plasma glucose levels showed a slight increase at *t*=+4 after ACTH injection, but FFA levels showed a large increase at *t*=+1 (×3.21,*FDR*<2.2*e*^−16^), with a strong variation across animals, and were almost back to basal levels at *t*=+4. The values measured at *t*=+1 were correlated with basal values (*r*^2^=0.42,*FDR*=2.30*e*^−06^). Cortisol induces a weak increase in circulating glucose and also potentiates the effect of other counter-regulatory hormones [24, 25] and increases FFA levels via acute lipolysis [26].

The data obtained here for clinical hematology measures are in the range of published values in pigs. These values show large variations with age and breed among other sources [27–29]. Although the total number of leucocytes was only marginally influenced by ACTH, large changes in leucocyte subpopulations were observed with an increase of the proportion of granulocytes and a decrease of the proportion of lymphocytes and monocytes at *t*=+1 and *t*=+4. These effects are consistent with the literature [30, 31] and result from the redistribution of leucocytes between blood and tissues [32]. Red blood cell related variables (red cells count, hematocrit and hemoglobin levels) were decreased after injection of ACTH and remained low at *t*=+24. Platelets were not influenced by injection of ACTH.

Sex did not influence any of the variables (*FDR*>0.05).

### Between-subject variability at *t*=0

**Fig. 2:**
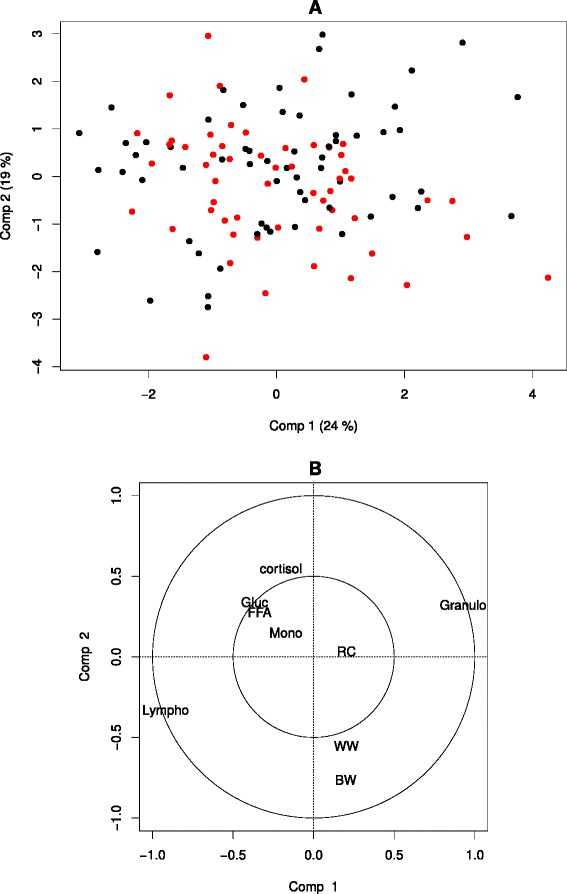
PCA on the biological variables identified as responding to ACTH, the birth and weaning weights at *t*=0. Colors symbolize the sex: Black = Female; Red = Male **a** Projection of the individuals on dimensions 1 and 2; **b** Projection of the variables on dimensions 1 and 2; BW = birth weight; WW = weaning weight; Lympho = lymphocyte ratio; Mono = monocytes ratio; Granulo = granulocyte ratio; RC = red cell count; Gluc = glucose; FFA = free fatty acids

Results of the PCA at *t*=0 on variables responding to ACTH and birth and weaning weights are shown in Fig. 2. As red cells count (RC), hematocrit (Hct) and hemoglobin (Hgb) are redundant variables, decision was made to keep only RC for the PCA. No outliers are identified on the projection of the individuals on the two first dimensions of the PCA. On this PCA, sex was not found to be related to the main variability (*i.e.*, to the first axes of the PCA) in the dataset. The first dimension shows an opposition between the proportion of granulocytes (positively correlated with this axis) and the proportion of lymphocytes (negatively correlated with this axis). The second axis shows an opposition between cortisol (positively correlated with this axis) and birth and (to a lesser extent) weaning weights (negatively correlated with this axis) with a strong opposition between these variables on the whole plan formed by the first and second axis. The other variables were not correlated with either of the first two dimensions of the PCA.

### Overall effect of the injection of exogenous ACTH on clinical biology variables

**Fig. 3:**
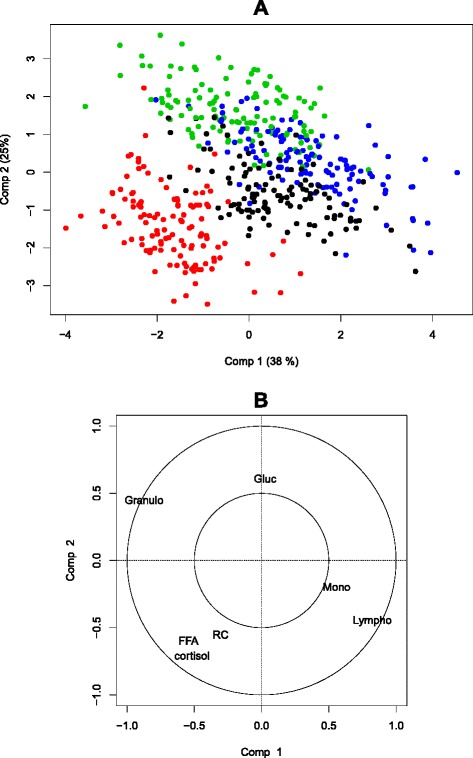
Multilevel PCA on the biological variables responding to ACTH. Colors symbolize the time of measurement; Black: *t*=0; Red: *t*=+1; Green: *t*=+4; Blue: *t*=+24; **a** Projection of the individuals on dimensions 1–2; **b** Projection of the variables on dimensions 1–2; Lympho = lymphocyte ratio; Mono = monocyte ratio; Granulo = granulocyte ratio; RC = red cell counts; Gluc = glucose; FFA = free fatty acids

Extraction of the within-subject data matrix prior to the application of a PCA analysis allows for the separation of the observations according to their time of measurement (see Fig. 3). The first component of the multi-level PCA opposes the observations at *t*=0 and *t*=+24 (positive coordinates on this axis) to the observations at *t*=+1 (negative coordinates on this axis), this time step corresponding to the peak of cortisol. The second component opposes the observations at *t*=+4 (positive coordinates on this axis) to the observations at *t*=+1 (negative coordinates on this axis). The representation of the variables shows that the first axis is mainly driven by an opposition between the proportion of granulocytes, FFA, cortisol and red cell count (high measures at *t*=+1), on one side, and lymphocytes and monocytes (high measures at *t*=0/+24), on the other side. The second axis shows an opposition between glucose (positively correlated with this axis, high measures at *t*=+4) and cortisol, FFA and red count (negatively correlated with this axis, high measures at *t*=+1).

### Specific links to the level of cortisol at *t*=+1

Correlations between cortisol at *t*=+1 and other variables at *t*=0 allows for the identification of variables which baseline levels may be directly or indirectly linked to the intensity of the cortisol level in response to ACTH, a measure of individual variation in HPA axis activity. Correlations are shown in Table 2. Only glucose and FFA levels at *t*=0 were significantly positively correlated with the level of cortisol at *t*=+1 (*FDR*<0.05). Correlations between cortisol at *t*=+1 and other variables at *t*=+1,*t*=+4 or *t*=+24 allows for the identification of variables which are directly linked to the level of cortisol when it reaches its peak during the stress response. FFA at *t*=+1 were positively correlated with cortisol at *t*=+1 and glucose at *t*=+1,*t*=+4 and *t*=+24 was negatively correlated with cortisol at *t*=+1 (*FDR*<0.05). No other variable was found to be significantly linked to the intensity of the cortisol level in response to ACTH.

**Table 2:** Correlation coefficients between the biological variables at *t*=0,*t*=+1,*t*=+4 and *t*=+24 and cortisol at *t*=+1 (*n* = 120)

Variables	*t*=0 (SE)	*t*=+1 (SE)	*t*=+4 (SE)	*t*=+24 (SE)
Free fatty acids	**0.35 (< 0.07)**	**0.45 (< 0.07)**	–0.13 (0.09)	0.04 (0.09)
Glucose	**0.30 (0.08)**	**–0.25 (0.08)**	**–0.27 (0.08)**	**–0.27 (0.08)**
Lymphocytes	–0.09 (0.09)	–0.10 (0.09)	–0.13 (0.09)	–0.07 (0.09)
Monocytes	–0.05 (0.09)	–0.02 (0.09)	–0.05 (0.09)	–0.04 (0.09)
Granulocytes	0.12 (0.09)	0.14 (0.09)	0.15 (0.09)	0.09 (0.09)
Red cells	0.06 (0.09)	0.07 (0.09)	–0.11 (0.09)	0.06 (0.09)
Hematocrit	0.06 (0.09)	0.13 (0.09)	–0.08 (0.09)	0.12 (0.09)
Hemoglobin	0.13 (0.09)	0.18 (0.08)	–0.15 (0.09)	0.03 (0.09)

*SE*: standard error of the correlation coefficient; **in bold**: significantly ≠0 (*F* *D* *R*<0.05)

### Differentially expressed genes

We used a comprehensive gene expression profiling by means of microarray analysis to identify clusters of genes differentially expressed in peripheral blood cells, taking into consideration the kinetics of the response with 4 time points (*t*∈{0,+1,+4,+24}). Differential analysis revealed 158 DE transcripts (adjusted *P*<0.05) matching 65 unique genes (The complete list with features is provided in Additional file 1). Among them, 23 genes were differentially expressed at *t*=+1 (5 down regulated/18 up-regulated), 25 were differentially expressed at *t*=+4 (8 down-regulated/17 up-regulated) and 17 were differentially expressed at *t*=+24 (all down-regulated). The only gene DE at both *t*=+1 and *t*=+4 was *SUCNR1* (Table 3). The adjusted *P*-values were smaller for tests between *t*=0 and *t*=+1 and between *t*=0 and *t*=+4 than between *t*=0 and *t*=+24 (see Additional file 2). This shows that the transcripts were more differentially expressed between *t*=0 and *t*=+1 and between *t*=0 and *t*=+4 than between *t*=0 and *t*=+24. Main effects of cortisol released by ACTH injection on gene expressions are thus observed at *t*=+1 and *t*=+4 with a return to baseline levels at *t*=+24.

**Table 3:** List of 65 unique genes differentially expressed in response to ACTH in pigs (*n*=30)

	Gene name	Adjusted P	Time point	Expression	Cluster
1	ADCY2	1.87E-03	1	UP-regulated	1
2	CEBPB	3.72E-09	1	UP-regulated	1
3	CEBPD	6.65E-07	1	UP-regulated	1
4	CPT1A	2.28E-06	1	UP-regulated	1
5	CXCR4	9.47E-06	1	UP-regulated	1
6	DDIT4	3.96E-07	1	UP-regulated	1
7	DUSP1	7.53E-03	1	UP-regulated	1
8	FKBP5	4.39E-06	1	UP-regulated	1
9	G30866	8.88E-05	1	UP-regulated	1
10	G39878	9.56E-04	1	UP-regulated	1
11	IL7R	6.63E-05	1	UP-regulated	1
12	MXD1	1.71E-03	1	UP-regulated	1
13	NFKBIA	2.72E-03	1	UP-regulated	1
14	PER1	9.37E-05	1	UP-regulated	1
15	PIK3IP1	2.89E-04	1	UP-regulated	1
16	RGS2	8.52E-08	1	UP-regulated	1
17	RHOB	4.08E-02	1	UP-regulated	1
18	TXNIP	1.78E-03	1	UP-regulated	1
19	ALOX5AP	1.20E-03	4	UP-regulated	2
20	ANG1	1.92E-02	4	UP-regulated	2
21	BASP1	4.11E-02	4	UP-regulated	2
22	C2H19orf59	1.06E-02	4	UP-regulated	2
23	CD14	3.99E-04	4	UP-regulated	2
24	CD24	1.82E-04	4	UP-regulated	2
25	CHI3L1	1.73E-02	4	UP-regulated	2
26	CHIT1	2.16E-02	4	UP-regulated	2
27	CLC4D	2.00E-03	4	UP-regulated	2
28	CRLD2	4.40E-02	4	UP-regulated	2
29	G42218	6.47E-03	4	UP-regulated	2
30	MEGF9	1.92E-04	4	UP-regulated	2
31	PDPN	2.08E-02	4	UP-regulated	2
32	RAB31	2.74E-02	4	UP-regulated	2
33	S100A12	5.47E-03	4	UP-regulated	2
34	S100A8	5.26E-03	4	UP-regulated	2
35	S100A9	2.92E-03	4	UP-regulated	2
36	CCL8	1.36E-04	1	DOWN-regulated	3
37	ALOX15	2.03E-07	4	DOWN-regulated	3
38	CAMK1	2.84E-09	4	DOWN-regulated	3
39	CSTA	9.20E-09	4	DOWN-regulated	3
40	FBP1	1.03E-04	4	DOWN-regulated	3
41	G36094	6.98E-10	4	DOWN-regulated	3
42	SLCO2B1	5.40E-13	4	DOWN-regulated	3
43	SUCNR1	2.58E-08	1 & 4	DOWN-regulated	3
44	CD79B	7.92E-04	1	DOWN-regulated	4
45	HHEX	3.32E-02	1	DOWN-regulated	4
46	MZB1	5.04E-03	1	DOWN-regulated	4
47	ST14	2.51E-02	1	DOWN-regulated	4
48	LOC396700	3.78E-06	4	DOWN-regulated	4
49	AKAP13	1.89E-02	24	DOWN-regulated	4
50	ARHGAP31	2.37E-03	24	DOWN-regulated	4
51	CLK1	2.52E-02	24	DOWN-regulated	4
52	DCAF15	1.56E-02	24	DOWN-regulated	4
53	FGR	6.95E-03	24	DOWN-regulated	4
54	G48605	8.08E-04	24	DOWN-regulated	4
55	HOPX	1.54E-02	24	DOWN-regulated	4
56	IGLV_7	3.07E-02	24	DOWN-regulated	4
57	LAS1L	4.12E-02	24	DOWN-regulated	4
58	LOC100626276	1.25E-03	24	DOWN-regulated	4
59	LOC396781	3.36E-02	24	DOWN-regulated	4
60	MAPK6	1.49E-03	24	DOWN-regulated	4
61	ORAI1	1.83E-03	24	DOWN-regulated	4
62	S100A1	5.43E-04	24	DOWN-regulated	4
63	TPST2	9.40E-04	24	DOWN-regulated	4
64	TRMT2A	3.61E-02	24	DOWN-regulated	4
65	XCL1	3.01E-02	24	DOWN-regulated	4

Genes are divided into clusters corresponding to the kinetics of the response to ACTH. Full description of the genes including their probe name and localisation is displayed in Additional file 1

**Fig. 4:**
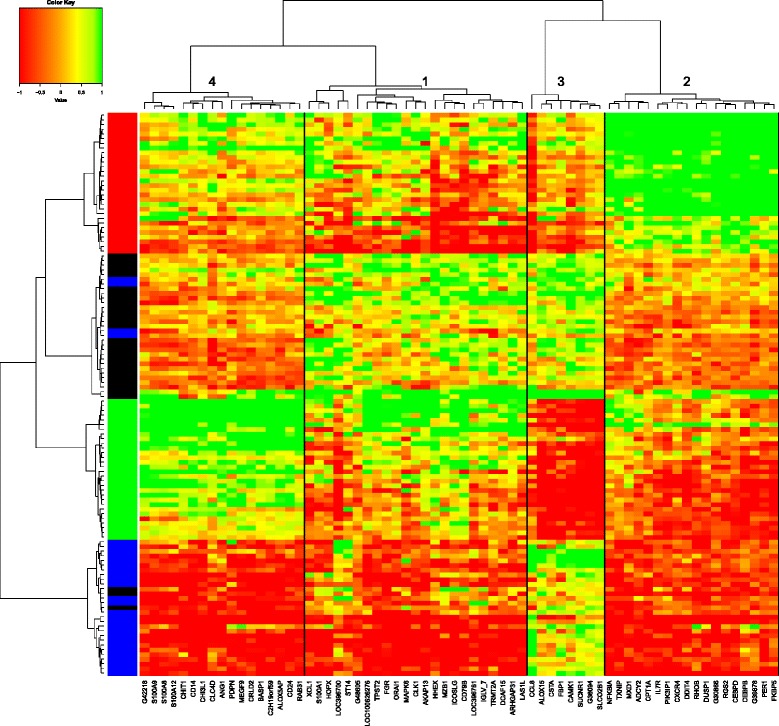
Hierarchical ascending classification of the 65 unique DE genes. A Ward method was used with an Euclidean distance matrix based on the correlations between genes. Genes are shown in column. Observations are shown in line with one line being a combination pig × time. Colors on the row dendrogram identify the time of measurement. Black: *t*=0; Red: *t*=+1; Green: *t*=+4; Blue: *t*=+24. Numbers on the column dendrogram identify each cluster

**Fig. 5:**
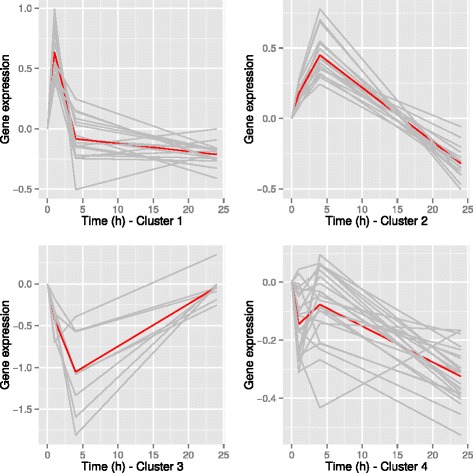
Average evolution of the genes in each of the cluster identified by the HAC on the 65 unique DE genes. Evolution of each gene is translated so that it is equal to 0 at *t*=0; Gray: Average evolution of each of the genes in the cluster. Red: Average evolution over all genes in the cluster (cluster 1: 18; cluster 2: 17; cluster 3: 8; cluster 4: 22)

HAC performed on the within-subject deviation matrix with the list of DE genes identified 4 groups of genes. Figure 4 shows that the 65 unique DE genes allow for an almost perfect classification of the observations with respect to their time of measurement. For every cluster, Fig. 5 shows the average evolution of each gene (gray) and the average evolution over the genes in the cluster (red).

Each cluster was then subjected to a functional analysis (results shown in Additional file 3). In each cluster, genes were DE (*FDR*<0.01) at each time point except for *t*=+24 in cluster 3 (*FDR*=0.57).

The first cluster (17 genes) was characterized by genes increasing with a peak of expression at *t*=1 and stable between *t*=+4 and *t*=+24. The DE genes of this cluster could be assembled into a functional network principally involved in neuroimmune functions. The present analysis reveals novel effects of ACTH on at least five genes related to immunoregulation (*FKBP5*, *IL7R*, *CEBPD*, *CEBPB* and *NFKB1A* in cluster 4). *FKBP5* (FK506 binding protein 51) is a decisive factor for the physiological stress response [33] and has an important role in stress-related phenotypes [34]. It modifies steroid hormone receptor sensitivity [35]. *CEBPB*, *DUSP1*, *FKBP5* and *NFKB1A* genes from this cluster are also involved in glucocorticoid receptor signaling. Glucocorticoids exert their classic anti-inflammatory role by acting on nearly all cell types of the immune system. The CCAAT/enhancer binding proteins (C/EBPs) are key regulators of cell differentiation and are also involved in the expression and production of inflammatory cytokines [36]. The increase of Period 1 gene (*PER1*) expression in peripheral blood cells by glucocorticoids was previously reported in humans [37]. Physical and inflammatory stressors induce the release of the adrenal glucocorticoid hormone that rapidly alter the expression of *PER1* in peripheral tissues through a GRE enhancer present in the gene promotor [38–40]. This gene is involved in the circadian rythm, in which the glucocorticoid mechanism plays a predominant role [41]. Another DE gene *DDIT4* (regulated in development and DNA damage response 1) was described as a surrogate biomarker of the efficiency of glucocorticoid receptor blockade in skeletal muscle [42]. Britto and collaborators showed that *DDIT4* expression was low under basal conditions but was highly increased in response to several catabolic stressors, like hypoxia and glucocorticoids [43]. Glucocorticoids were shown to up-regulate *DUSP1* in peripheral tissues [44] but constrain the increase of *DUSP1* gene expression in the central components of the HPA axis [45]. In vitro studies have shown that glucocorticoid suppression of some MAP-kinase dependent cellular processes depends on glucocorticoid mediated up-regulation of *DUSP1* gene expression [46].

The second cluster (17 genes) was characterized by genes with an increase between *t*=0 and *t*=+4 and a decrease between *t*=+4 and *t*=+24. This cluster with genes up-regulated at *t*=+4 is largely related to biological processes such as inflammatory and immune response and genes of which products are located in the plasma membrane. Among these genes, two are particularly interesting. *CD14* gene is a component of the innate immune system and has been shown to be sensitive to stress in pigs [47]. *MEGF9* gene was shown to be induced by cortisol in human fetal cells in vitro [48].

The third cluster (8 genes) includes the genes decreasing between *t*=0 and *t*=+4 and returning to a basal level between *t*=+4 and *t*=+24. No ontology was significantly enriched by genes of this cluster. It is interesting to underline here the *ALOX15* gene (arachidonate 15-lipoxygenase) which is a member of the ALOX family and related to cancer and immune responses. This gene was also reported as a dexamethasone-responsive gene with nearby glucocorticoid receptor-binding sites [49].

The genes related to the fourth cluster (22 genes) decrease between *t*=0 and *t*=+1, increase between *t*=+1 and *t*=+4 and decrease between *t*=+4 and *t*=+24. The fourth cluster corresponds to genes with an overall expression decreasing between *t*=0 and *t*=+24. They are significantly linked to biological processes such as protein phosphorylation and kinase activity. Among the genes involved in this cluster *ARHGAP31* and *ARHGAP* family genes were found to be differentially expressed in macrophages treated with dexamethasone [50, 51].

### Integration of biological and gene expression data

All DE genes were found significantly differentially expressed over time in the mixed model described in Section “Integration” (*FDR*<0.05). These genes are thus differentially expressed over time even when adjusting for changes in white cells populations. Among them, 34 genes had their expression significantly negatively influenced by L/G ratio (see Additional file 1), meaning that these genes are over-expressed when L/G ratio decreases. Genes with a significant effect of L/G ratio were mainly identified as genes of cluster 2, over-expressed at *t*=+4 (17/17) and cluster 1, over-expressed at *t*=+1 (12/18) and to a lesser extent as genes of cluster 4, under-expressed at *t*=+24 (5/22). No gene of cluster 3 was significantly explained by L/G ratio. Results of the functional analysis of this list of 34 genes are shown in Additional file 4. Biological functions significantly enriched include regulation of apoptotic process, response to lipopolysaccharide, inflammatory and innate immune response, defense response to bacterium and positive regulation of NF-kappaB transcription factor activity.

**Fig. 6:**
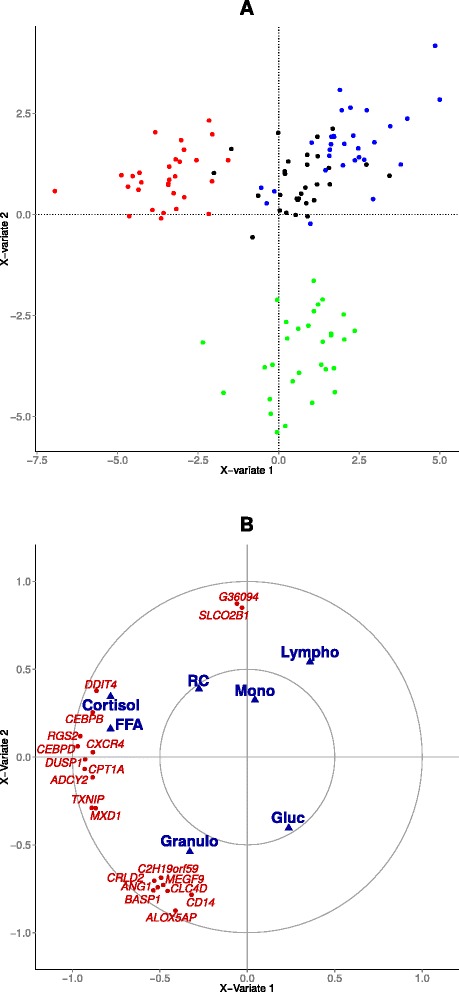
PLS regression predicting the biological variables responding to ACTH from the DEG expression **a** projection of the observations with one point being a combination: time × pig; Black: *t*=0; Red: *t*=+1; Green: *t*=+4; Blue: *t*=+24. **b** projection of the variables; Blue: biological variables; Red: gene expressions; 10 genes/components were kept using a sparse approach

The 65 DE genes and the biological variables were then subjected to a multilevel PLS. Figure 6 shows that the first axis of the multilevel PLS opposes observations at *t*=+1 after injection to all others, while the second axis opposes observations at *t*=+4*vs.* all others, similarly as what was already established in multi-level PCA of the biological variables in Section “Overall effect of the injection of exogenous ACTH on clinical biology variables”.

On the first axis, cortisol and FFA levels are strongly positively correlated with the expressions of *CEBPB*, *RGS2*, *RHOB*, *PER1*, *FKBP5*, *CEBPD*, *DDIT4*, *CPT1A* and *DUSP1*. All these genes belong to the first cluster identified earlier and are linked to molecular functions such as protein binding and transcription regulation.

The second axis of the multilevel PLS is characterized by the opposition between the proportion of lymphocytes and monocytes *vs.* the proportion of granulocytes. This axis is positively correlated with *SUCNR1*, *SLCO2B1*, *FBP1* and *LOC396700*. These genes belong to the third cluster and are related to glycolysis and glycogenesis. *SUCNR1* (succinate receptor 1) is decreased at *t*=+1 and increased at *t*=+4. Succinate has a wide range of metabolic actions and regulates the functions of macrophages [52]. The axis is negatively correlated with *CD14*, *CLC4D*, *CHIT1*, *MEGF9* and *C2H19orf59*. These genes belong to the second cluster which is linked to molecular functions such as inflammatory response, but their relationships with cortisol or stress are not yet clearly established.

**Fig. 7:**
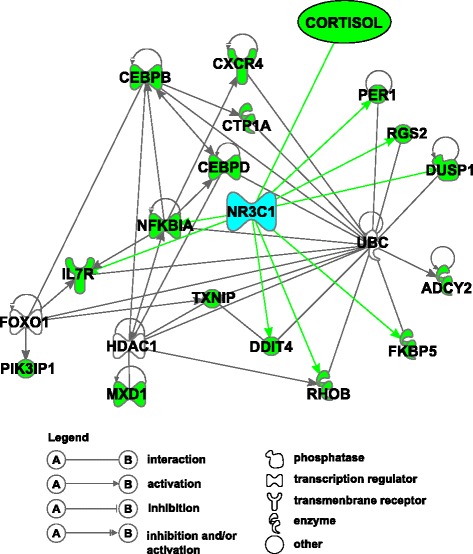
Gene network related to glucocorticoid response in whole blood transcriptome 1 h after ACTH injection This network corresponds to the genes up-regulated 1 h after ACTH injection (cluster 1, green nodes). It combines bibliographic (best enrichment score network =45) and regulatory relationships (genes co-regulated by the same regulator in blue with the highest enrichment score (*p*-value=2.00*E*−09), green lines) proposed by Ingenuity software. Cortisol has the highest plasma level at *t*=+1 and acts through the glucocorticoid receptor NR3C1

Eight genes (*DDIT4*, *DUSP1*, *FKBP5*, *IL7R*, *NFKBIA*, *PER1*, *RGS2*, *RHOB*, Fig. 7) are functionally connected to each other by NR3C1. The NR3C1 (nuclear receptor subfamily 3, group C, member 1) is the glucocorticoid receptor, which can function both as a transcription factor that binds to glucocorticoid response elements in the promoters of glucocorticoid responsive genes, and as a regulator of other transcription factors. Functional consequences of glucocorticoid receptor polymorphisms were reported in pigs [9–13]. Mutations in *NR3C1* have been previously demonstrated to be associated with generalized glucocorticoid resistance [53]. It is interesting to highlight the DE genes that encode transcription factors. They play a crucial role in regulating gene expression and are fit to regulate diverse cellular processes by interacting with other proteins. Most of them have not yet been described as important in transcription networks involved in stress responses. If the genes are co-expressed it is highly probable that they are co-regulated. This knowledge can provide new patterns of biomarkers of the individual sensitivity to cortisol that is our field of interest in this study.

Our results are in accordance with several studies on the effects of glucocorticoid hormones on peripheral blood cells. Numerous genes related to cluster 1 and shown as ACTH responsive were found differentially expressed in stress-related investigations. Five genes found in our study (*CXCR4*, *DUSP1*, *FKBP5*, *IL7R*, *TXNIP*) were proposed as markers of differential glucocorticoid sensitivity [54, 55]. *NFKBIA*, *DUSP1*, *CEBPD*, *FKBP5* genes were also found to be associated with up- and down-regulated clusters in response to continuous 24 h cortisol infusion [56]. Ponsuksili and collaborators [57] describe *NFKBIA*, *CEBPB* and *CEBPD* as genes of which hepatic expression levels are correlated with plasma cortisol concentrations. Up-regulation of *PER1* gene upon GR activation was confirmed by genome-wide study of glucocorticoid receptor binding sites in neuronal PC12 cells [58]. However, *DDIT4* was shown to be down-regulated by GR activation rather than up-regulated in this analysis.

While looking for genes of which expressions at *t*=0,*t*=+1,*t*=+4 or *t*=+24 were significantly correlated with the level of cortisol at *t*=+1, only two genes were identified: *TRMT2A* (*FDR*=0.04), a gene involved in the methylation of tRNA, and *LOC100626276* (*FDR*=0.04), a gene of which function has not been identified yet. There is a negative relationship between the expression of cortisol at *t*=+1 and the expression of these two genes at *t*=0 (−0.45 and −0.63 for *TRMT2A* and *LOC100626276*, respectively). This implies that when their baseline expression is higher, the intensity of the cortisol response to ACTH decreases.

## Conclusions

The present work shows the interest of transcriptomic data analysis at multiple levels. In other studies, genetic markers found through an analysis of transcription factor binding sites of differentially expressed genes in peripheral blood cells have been proposed in humans to identify the chronic stress related to psychopathological conditions [59, 60]. In farm animals, this approach was used in horses [61]. These studies show chronic stress-related changes in the balance between the expression of stress-related genes regulated by glucocorticoids and those regulated by inflammation-related factors. Furthermore, recent data in humans show that the immune system function can also be assessed through blood transcriptomics in health and disease [62].

In the present study, we identified 65 genes differentially expressed in peripheral blood cells of pigs in response to ACTH at different times after injection. It therefore supplies biological markers of HPA axis activation at the gene expression level, and the knowledge on functional gene clusters will help to elucidate the biological processes involved. Moreover, these genes might be candidates for a better understanding of the molecular mechanisms related to stress responses. Thus, blood transcriptome analysis appears as a promising avenue to develop multidimensional biological markers related to robustness. These markers should be used in the study of the genetic mechanisms of adaptation in farm animals that will help to deliver genetic strategies to animal breeders in order to balance production objectives and robustness of animals as well as their welfare [2].

## Availability of supporting data

The data sets supporting the results of this article are available in Gene Expression Omnibus (GEO repository, http://www.ncbi.nlm.nih.gov/geo/,) through the accession number GSE71207).

## Additional files

Additional file 1 ∙ Gene name: Name of the gene ∙ Probe Name, Agilent GPL16524: Probe names used on the microarray Agilent GPL16524 ∙ L/G coefficient: L/G ratio coefficient estimate ∙ Adjusted pval (LG): adjusted *P*-value associated with the L/G ratio coefficient ∙ adj.pval: adjusted *P*-value of the test at the time measurement where the most significant duplicate of the gene is DE ∙ Time point: time measurement where the gene is DE ∙ Expression: whether the DEG is up or down-regulated ∙ Cluster: cluster in which the gene is classified by HAC ∙ Gene description: informations on the gene’s molecular function ∙ Location: chromosomal location of the gene. (XLS 23 kb)

Additional file 2
**Distribution of the rank of the significant adjusted** ***P*** **-values in the tests for DE transcripts between** ***t*** **= 0 and** ***t*** **= + 1,** ***t*** **= 0 and** ***t*** **= + 4 and** ***t*** **= 0 and** ***t*** **= + 24 ‘.pdf’ file.** *P*-values are smaller at *t*=+1 and *t*=+4 than at *t*=+24 implying that the transcripts were overall more differentially expressed between *t*=0 and *t*=+1 and between *t*=0 and *t*=+4 than between *t*=0 and *t*=+24. (PDF 4 kb)

Additional file 3
**Complete list of enriched GO (Biological process (BP), Molecular function (MF) and Cellular Component (CC) for each of the cluster identified with the hierarchical ascending clustering ‘.xls’ file.** Features the GO items, the corresponding functions, the class of ontology, the number of genes in the input list (enriching a GO and total number) and in the reference list (enriching a GO and total number), the raw and the adjusted Fisher’s exact test *P*-value and the list of genes. (XLS 10 kb)

Additional file 4
**Complete list of enriched GO (Biological process (BP), Molecular function (MF) and Cellular Component (CC) for 34 genes for which L/G ratio had a significant effect ‘.xls’ file.** Features the GO items, the corresponding functions, the class of ontology, the number of genes in the input list (enriching a GO and total number) and in the reference list (enriching a GO and total number), the raw and the 679 adjusted Fisher’s exact test *P*-value and the list of genes. (XLS 7 kb)

## Abbreviations

ACTH: adrenocorticotropic hormone
BH: Benjamini-Hochberg
BW: birth weight
CBG: corticosteroid binding globulin
DE: differentially expressed
FDR: false discovery rate
GR: glucocorticoid receptor
GRE: Glucocorticoid response element
HAC: Hierarchical ascendant classification
Hct: Hematocrit
Hgb: Hemoglobin
HPA: Hypothalamic-pituitary-adrenocortical
L/ G: Lymphocytes/ granulocytes
PCA: prinicpal component analysis
PLS: partial least square regression
RC: red cells
SEM: standar error of the mean
WW: weaning weight

## References

[1] Rauw W, Kanis E, Noordhuizen-Stassen E, Grommers F (1998). Undesirable side effects of selection for high production efficiency in farm animals: a review. Livest Prod Sci.

[2] Mormede P, Terenina E (2012). Molecular genetics of the adrenocortical axis and breeding for robustness. Domest Anim Endocrinol.

[3] Mormède P, Foury A, Terenina E, Knap P (2011). Breeding for robustness: the role of cortisol. Animal.

[4] Mormede P, Foury A, Barat P, Corcuff JB, Terenina E, Marissal-Arvy N (2011). Molecular genetics of hypothalamic–pituitary–adrenal axis activity and function. Ann N Y Acad Sci.

[5] Désautés C, Bidanel J, Milan D, Iannuccelli N, Amigues Y, Bourgeois F (2002). Genetic linkage mapping of quantitative trait loci for behavioral and neuroendocrine stress response traits in pigs. J Anim Sci.

[6] Larzul C, Terenina E, Foury A, Billon Y, Louveau I, Merlot E, et al.The cortisol response to ACTH in pigs, heritability and influence of corticosteroid-binding globulin. 2015. In press. 10.1017/S1751731115001767 26302113

[7] Hazard D, Liaubet L, SanCristobal M, Mormède P (2008). Gene array and real time pcr analysis of the adrenal sensitivity to adrenocorticotropic hormone in pig. BMC genomics.

[8] Necela BM, Cidlowski JA (2004). Mechanisms of glucocorticoid receptor action in noninflammatory and inflammatory cells. Ann Am Thorac Soc.

[9] Murani E, Reyer H, Ponsuksili S, Fritschka S, Wimmers K (2012). A substitution in the ligand binding domain of the porcine glucocorticoid receptor affects activity of the adrenal gland. PLoS ONE.

[10] Yang X, Liu R, Albrecht E, Dong X, Maak S, Zhao R (2012). Breed-specific patterns of hepatic gluconeogenesis and glucocorticoid action in pigs. Archiv Tierzucht.

[11] Reyer H, Ponsuksili S, Wimmers K, Murani E (2013). Transcript variants of the porcine glucocorticoid receptor gene (nr3c1). Gen Comp Endocrinol.

[12] Reyer H, Ponsuksili S, Wimmers K, Murani E (2014). Association of n-terminal domain polymorphisms of the porcine glucocorticoid receptor with carcass composition and meat quality traits. Anim Genet.

[13] Terenina E, Babigumira BM, Le Mignon G, Bazovkina D, Rousseau S, Salin F (2013). Association study of molecular polymorphisms in candidate genes related to stress responses with production and meat quality traits in pigs. Domest Anim Endocrinol.

[14] Torgo L (2010). Data Mining with R: Learning with Case Studies. Chapman & Hall/CRC Data Mining and Knowledge Discovery Series.

[15] Voillet V, SanCristobal M, Lippi Y, Martin PG, Iannuccelli N, Lascor C (2014). Muscle transcriptomic investigation of late fetal development identifies candidate genes for piglet maturity. BMC Genom.

[16] Bolstad BM, Irizarry RA, Åstrand M, Speed TP (2003). A comparison of normalization methods for high density oligonucleotide array data based on variance and bias. Bioinformatics.

[17] R Core Team (2014). R: A language and environment for statistical computing.

[18] Liquet B, Lê Cao KA, Hocini H, Thiébaut R (2012). A novel approach for biomarker selection and the integration of repeated measures experiments from two assays. BMC Bioinform.

[19] Saporta G (2011). Probabilités, Analyse des Données et Statistique.

[20] Lê Cao KA, Rossouw D, Robert-Granié C, Besse P (2008). A sparse pls for variable selection when integrating omics data. Stat Appl Genet Mol Biol.

[21] Benjamini Y, Hochberg Y (1995). Controlling the false discovery rate: a practical and powerful approach to multiple testing. J Roy Stat Soc B Met..

[22] Smedley D, Haider S, Durinck S, Pandini L, Provero P, Allen J (2015). The biomart community portal: an innovative alternative to large, centralized data repositories. Nucleic Acids Res.

[23] Hennessy D, Stelmasiak T, Johnston N, Jackson P, Outch K (1988). Consistent capacity for adrenocortical response to acth administration in pigs. Am J Vet Res.

[24] Eigler N, Saccà L, Sherwin RS (1979). Synergistic interactions of physiologic increments of glucagon, epinephrine, and cortisol in the dog: a model for stress-induced hyperglycemia. J Clin Invest.

[25] Shamoon H, Hendler R, Sherwin RS (1981). Synergistic interactions among antiinsulin hormones in the pathogenesis of stress hyperglycemia in humans. J Clin Endocr Metab.

[26] Peckett AJ, Wright DC, Riddell MC (2011). The effects of glucocorticoids on adipose tissue lipid metabolism. Metabolism.

[27] Flori L, Gao Y, Laloë D, Lemonnier G, Leplat JJ, Teillaud A (2011). Immunity traits in pigs: substantial genetic variation and limited covariation. PLoS One.

[28] Sutherland M, Rodriguez-Zas S, Ellis M, Salak-Johnson J (2005). Breed and age affect baseline immune traits, cortisol, and performance in growing pigs. J Anim Sci.

[29] Friendship R, Lumsden J, McMillan I, Wilson M (1984). Hematology and biochemistry reference values for ontario swine. Can J Comparat Med.

[30] Wallgren P, Wilén IL, Fossum C (1994). Influence of experimentally induced endogenous production of cortisol on the immune capacity in swine. Vet Immunol Immunop.

[31] Salak-Johnson JL, McGlone JJ, Norman RL (1996). In vivo glucocorticoid effects on porcine natural killer cell activity and circulating leukocytes. J Anim Sci.

[32] Dhabhar FS (2002). Stress-induced augmentation of immune function—the role of stress hormones, leukocyte trafficking, and cytokines. Brain Behav Immun.

[33] Touma C, Gassen NC, Herrmann L, Cheung-Flynn J, Büll DR, Ionescu IA (2011). Fk506 binding protein 5 shapes stress responsiveness: modulation of neuroendocrine reactivity and coping behavior. Biol Psychiatry.

[34] Binder EB (2009). The role of fkbp5, a co-chaperone of the glucocorticoid receptor in the pathogenesis and therapy of affective and anxiety disorders. Psychoneuroendocrinology.

[35] Storer CL, Dickey CA, Galigniana MD, Rein T, Cox MB (2011). Fkbp51 and fkbp52 in signaling and disease. Trends Endocrinol Metab.

[36] Cloutier A, Guindi C, Larivée P, Dubois CM, Amrani A, McDonald PP (2009). Inflammatory cytokine production by human neutrophils involves c/ebp transcription factors. J Immunol.

[37] Cuesta M, Cermakian N, Boivin DB (2015). Glucocorticoids entrain molecular clock components in human peripheral cells. FASEB J.

[38] Takahashi S, Yokota S-i, Hara R, Kobayashi T, Akiyama M, Moriya T (2001). Physical and inflammatory stressors elevate circadian clock gene mper1 mrna levels in the paraventricular nucleus of the mouse. Endocrinology.

[39] Hida A, Koike N, Hirose M, Hattori M, Sakaki Y, Tei H (2000). The human and mouse period1 genes: five well-conserved e-boxes additively contribute to the enhancement of mper1 transcription. Genomics.

[40] Yamamoto T, Nakahata Y, Tanaka M, Yoshida M, Soma H, Shinohara K (2005). Acute physical stress elevates mouse period1 mrna expression in mouse peripheral tissues via a glucocorticoid-responsive element. J Biol Chem.

[41] Burioka N, Takata M, Endo M, Miyata M, Takeda K, Chikumi H (2007). Treatment with *β*2-adrenoceptor agonist in vivo induces human clock gene, per1, mrna expression in peripheral blood. Chronobiol Int.

[42] Kumari R, Willing LB, Jefferson LS, Simpson IA, Kimball SR (2011). Redd1 (regulated in development and dna damage response 1) expression in skeletal muscle as a surrogate biomarker of the efficiency of glucocorticoid receptor blockade. Biochem Biophys Res Commun.

[43] Britto FA, Begue G, Rossano B, Docquier A, Vernus B, Sar C (2014). Redd1 deletion prevents dexamethasone-induced skeletal muscle atrophy. Am J Physiol Endocrinol Metab.

[44] Clark AR, Martins JRS, Tchen CR (2008). Role of dual specificity phosphatases in biological responses to glucocorticoids. J Biol Chem.

[45] Osterlund CD, Thompson V, Hinds L, Spencer RL (2014). Absence of glucocorticoids augments stress-induced mkp1 mrna expression within the hypothalamic–pituitary–adrenal axis. J Endocrinol.

[46] Burke SJ, Goff MR, Updegraff BL, Lu D, Brown PL, Minkin Jr SC, et al.Regulation of the ccl2 gene in pancreatic *β*-cells by il-1 *β* and glucocorticoids: role of journal=mkp-1, PLoS ONE. 2012; 7(10):e46986. 10.1371/journal.pone.0046986 PMC3467264 23056550

[47] Oster M, Muráni E, Ponsuksili S, Richard B, Turner SP, Evans G (2014). Transcriptional responses of pbmc in psychosocially stressed animals indicate an alerting of the immune system in female but not in castrated male pigs. BMC Genom.

[48] Salaria S, Chana G, Caldara F, Feltrin E, Altieri M, Faggioni F (2006). Microarray analysis of cultured human brain aggregates following cortisol exposure: Implications for cellular functions relevant to mood disorders. Neurobiol Dis.

[49] Reddy TE, Gertz J, Crawford GE, Garabedian MJ, Myers RM (2012). The hypersensitive glucocorticoid response specifically regulates period 1 and expression of circadian genes. Mol Cell Biol.

[50] Uhlenhaut NH, Barish GD, Ruth TY, Downes M, Karunasiri M, Liddle C (2013). Insights into negative regulation by the glucocorticoid receptor from genome-wide profiling of inflammatory cistromes. Mol Cell.

[51] So A, Chaivorapol C, Bolton EC, Li H, Yamamoto KR (2007). Determinants of cell-and gene-specific transcriptional regulation by the glucocorticoid receptor. PLoS Genet.

[52] Mills E, O’Neill LA (2014). Succinate: a metabolic signal in inflammation. Trends Cell Biol.

[53] Donner KM, Hiltunen TP, Jänne OA, Sane T, Kontula K (2013). Generalized glucocorticoid resistance caused by a novel two-nucleotide deletion in the hormone-binding domain of the glucocorticoid receptor gene nr3c1. Eur J Endocrinol.

[54] Donn R, Berry A, Stevens A, Farrow S, Betts J, Stevens R (2007). Use of gene expression profiling to identify a novel glucocorticoid sensitivity determining gene, bmprii. FASEB J.

[55] Menke A, Arloth J, Pütz B, Weber P, Klengel T, Mehta D (2012). Dexamethasone stimulated gene expression in peripheral blood is a sensitive marker for glucocorticoid receptor resistance in depressed patients. Neuropsychopharmacology.

[56] Kamisoglu K, Sleight K, Nguyen TT, Calvano SE, Coyle SM, Corbett SA (2014). Effects of coupled dose and rhythm manipulation of plasma cortisol levels on leukocyte transcriptional response to endotoxin challenge in humans. Innate Immun.

[57] Ponsuksili S, Du Y, Murani E, Schwerin M, Wimmers K (2012). Elucidating molecular networks that either affect or respond to plasma cortisol concentration in target tissues of liver and muscle. Genetics.

[58] Polman JAE, Welten JE, Bosch DS, de Jonge RT, Balog J, van der Maarel SM (2012). A genome-wide signature of glucocorticoid receptor binding in neuronal pc12 cells. BMC Neurosci.

[59] Cole SW (2010). Elevating the perspective on human stress genomics. Psychoneuroendocrinology.

[60] O’Donovan A, Sun B, Cole S, Rempel H, Lenoci M, Pulliam L (2011). Transcriptional control of monocyte gene expression in post-traumatic stress disorder. Dis Markers.

[61] Lansade L, Valenchon M, Foury A, Neveux C, Cole SW, Layé S (2014). Behavioral and transcriptomic fingerprints of an enriched environment in horses (equus caballus). PloS one.

[62] Chaussabel D, Pascual V, Banchereau J (2010). Assessing the human immune system through blood transcriptomics. BMC Biol.

