# Supplementary figures and images for: Time course of the response to ACTH in pig: biological and transcriptomic study
Source: BMC Genomics. 2015 Nov 17;16:961. doi: 10.1186/s12864-015-2118-8 (PMC4650497; doi:10.1186/s12864-015-2118-8)

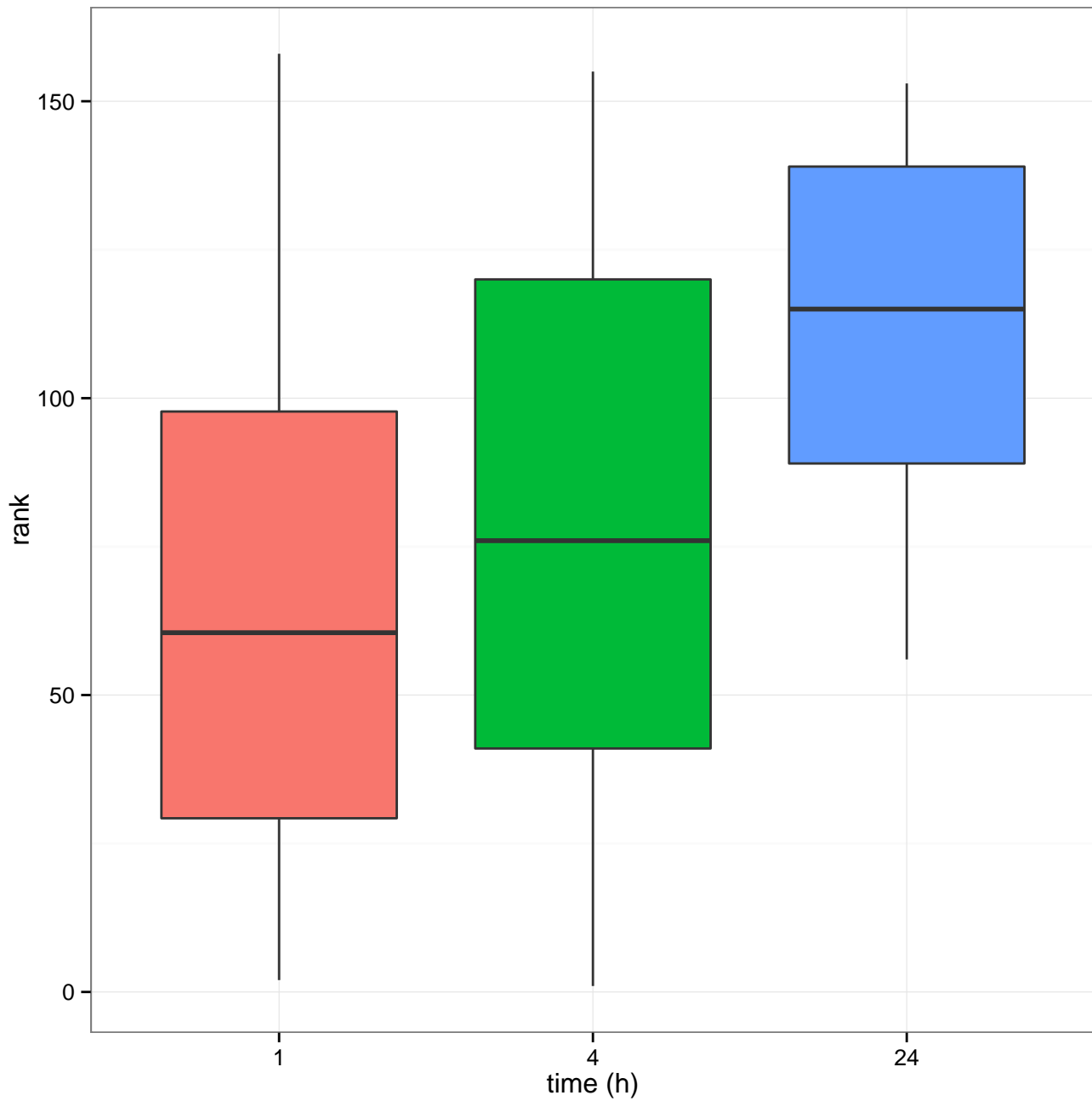

Supplement: Additional file 2 — Distribution of the rank of the significant adjusted P -values in the tests for DE transcripts between t = 0 and t = + 1, t = 0 and t = + 4 and t = 0 and t = + 24 ‘.pdf’ file. P-values are smaller at t=+1 and t=+4 than at t=+24 implying that the transcripts were overall more differentially expressed between t=0 and t=+1 and between t=0 and t=+4 than between t=0 and t=+24. (PDF 4 kb) [file 12864_2015_2118_MOESM2_ESM.pdf]
